# Cannabis-Associated Spontaneous Coronary Artery Dissection in a Community Hospital: A Case-Based Focused Literature Review

**DOI:** 10.7759/cureus.103651

**Published:** 2026-02-15

**Authors:** Sebastian Hernandez Mejia, Karan B Bhanushali, Rushaniya A Umyarova, Nay Htyte

**Affiliations:** 1 Internal Medicine/Cardiology, Richmond University Medical Center, New York City, USA; 2 Medicine, Rural Medical College, Pravara Institute of Medical Sciences, Loni, IND; 3 Internal Medicine, Roger Williams Medical Center, Providence, USA; 4 Internal Medicine, Richmond University Medical Center, New York City, USA; 5 Cardiovascular Medicine, Richmond University Medical Center, New York City, USA

**Keywords:** ecg localization, left circumflex artery dominance, multivessel scad, scad and cannabis, scad diagnosis, scad management, spontaneous coronary artery dissection

## Abstract

This case report highlights a rare and diagnostically challenging presentation of spontaneous coronary artery dissection (SCAD) in a healthy 39-year-old woman, with symptoms beginning while smoking cannabis after heavy recent use. It also underscores real-world limitations in community settings where advanced intracoronary imaging may be unavailable or unsuitable and where diagnosis and management rely on angiographic morphology and clinical context. In our patient, angiography localized the culprit to the distal left circumflex (LCx) artery, with minimal atherosclerotic burden elsewhere. Extracoronary imaging was negative for fibromuscular dysplasia (FMD), and follow-up CT coronary angiography demonstrated interval healing features that strengthened diagnostic confidence compared with many prior published cannabis-related cases. The favorable outcome with conservative therapy aligns with contemporary recommendations for stable SCAD, supporting noninterventional strategies when high-risk features are absent. Overall, this report adds to the limited literature linking cannabis exposure to SCAD and highlights cannabis as a possible, under-recognized trigger warranting greater clinician awareness and further investigation.

## Introduction

Spontaneous coronary artery dissection (SCAD) is an uncommon cause of acute coronary syndrome (ACS). Increasing recognition in younger women with few traditional atherosclerotic risk factors, together with its potential for significant morbidity, has raised awareness of SCAD over the last decade. First described in 1931, SCAD is now recognized as an important etiology of ACS, although its true incidence remains uncertain and is estimated at approximately 0.1%-4% of ACS presentations [1-5].

Patient-centered research in recent years has improved understanding of SCAD risk factors, refined diagnostic recognition (including SCAD-specific angiographic classification), and, importantly, shifted acute and long-term management toward conservative strategies in stable patients [6-19]. This is particularly relevant because, in routine practice, diagnosis still relies on angiography and clinical context, and intracoronary imaging is not universally available.

Cannabis use has been increasingly linked to cardiovascular events, yet SCAD temporally associated with cannabis exposure remains uncommon. Published evidence is limited to case reports and small series. Across reports of cannabis-associated SCAD and coronary dissection, systematic evaluation for alternative etiologies (especially fibromuscular dysplasia (FMD)), intracoronary imaging, and follow-up coronary imaging are inconsistently documented [20-25]. Because SCAD often coexists with underlying arteriopathies, comprehensive assessment strengthens diagnostic confidence and improves interpretation.

We present one of the few reported cases of angiographically diagnosed SCAD temporally associated with heavy cannabis use, managed in a community hospital without access to intracoronary imaging. The case featured discordant findings across initial diagnostic testing. Most importantly, we analyze the clinical reasoning behind the diagnosis with a focused, evidence-based literature review. Finally, we propose two plausible explanations for the observed discrepancies, including emerging evidence that left circumflex (LCx) ischemia may be under-recognized on the 12-lead ECG, supported by interval healing on follow-up coronary CT angiography.

## Case presentation

**Figure 1:**
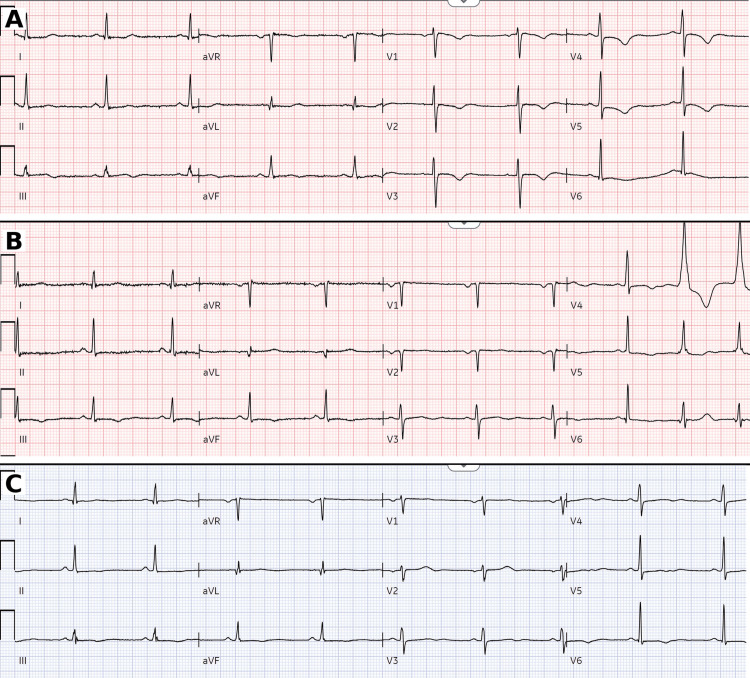
Serial ECGs (A) ECG on presentation showed anterolateral ST-segment depression with early dynamic ST-T changes. (B) Repeat ECG during clinical evolution demonstrated persistent anterolateral ST-segment depression with evolving precordial T-wave inversion, consistent with ongoing ischemia. (C) Follow-up ECG after stabilization showed improvement/resolution of prior ischemic changes with nonspecific ST-T abnormalities.

A 39-year-old woman with no prior chest symptoms and no traditional cardiovascular risk factors presented with new-onset, typical anginal chest pain radiating to the left arm, associated with diaphoresis, nausea, and vomiting, occurring at rest. She reported two similar, self-limited episodes earlier that day while smoking cannabis, each resolving with rest. She endorsed chronic smoked cannabis use with a marked recent increase in frequency and amount, including heavy use the morning of presentation. Medication history included prior oral contraceptive use, discontinued one month prior to presentation. She also reported daily alcohol intake (two glasses of wine). Pregnancy testing was negative; she was not peripartum (last delivery three years earlier) and denied acute emotional stress at symptom onset. On arrival, she was hemodynamically stable and pain-free after aspirin 324 mg and sublingual nitroglycerin 0.4 mg. Physical examination was unremarkable. ECG demonstrated anterolateral ST-segment depression with dynamic T-wave changes (Figure 1A). High-sensitivity troponin was elevated at 450 ng/L (reference 0-34 ng/L). Urine toxicology was positive for cannabinoids and negative for cocaine and other recreational drugs.

Serial evaluation showed a rapid rise in high-sensitivity troponin from 450 ng/L to 18,000 ng/L, paired with persistent anterolateral ST depression and evolving precordial T-wave inversion (Figure 1B). She was treated as a non-ST-elevation myocardial infarction (NSTEMI) with ticagrelor loading and an unfractionated heparin infusion. Troponin subsequently peaked at 59,000 ng/L before downtrending; the corresponding ECG demonstrated nonspecific T-wave abnormalities (Figure 1C). Transthoracic echocardiography showed low-normal left ventricular ejection fraction (LVEF) (50%-55%) with inferior wall hypokinesis and mild mitral regurgitation. Given the rapid troponin rise with evolving ECG changes and discordant echocardiographic findings, she was transferred urgently for coronary angiography.

**Figure 2:**
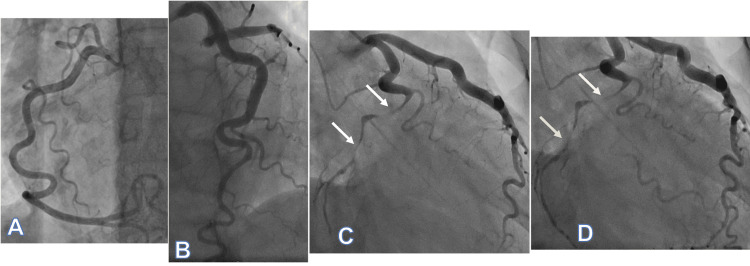
Coronary angiography Coronary angiography demonstrated (A) a tortuous RCA without significant atherosclerotic disease and (B) LAD artery without obstructive disease. (C) Early injection of the left system showed severe focal-to-diffuse long-segment narrowing of the distal LCx beyond the first obtuse marginal branch (arrows). (D) Delayed/late images demonstrated late filling of the distal LCx artery beyond the narrowing via collateral flow (arrows). RCA: right coronary artery; LAD: left anterior descending; LCx: left circumflex

Coronary angiography demonstrated a tortuous right coronary artery (RCA) with smooth, symmetric curvature and no significant atherosclerotic disease (Figure 2A). The left anterior descending (LAD) artery was also free of obstructive disease (Figure 2B). In contrast, the LCx artery showed severe focal-to-diffuse high-grade narrowing of the distal segment beyond the first obtuse marginal branch, with delayed distal opacification predominantly via collateral flow (Figures 2C-2D). The differential diagnosis included atherothrombotic occlusion (plaque rupture with thrombus), coronary embolism, vasospasm, and coronary dissection. Intracoronary imaging (optical coherence tomography (OCT)/intravascular ultrasound (IVUS)) was not performed (OCT unavailable; IVUS unsuitable for distal LCx assessment); diagnosis therefore relied on angiographic features and clinical context.

Angiography did not show a discrete intimal flap or contrast staining to definitively confirm dissection; instead, the dominant finding was diffuse narrowing of the distal LCx. The left main and left anterior descending arteries showed no significant disease to account for the evolving ECG changes. Given the angiographic appearance, overall clinical context, and the patient’s stability, the team favored SCAD and pursued conservative management without intervention. She remained pain-free and hemodynamically stable, with low-normal LVEF and resolution of ECG abnormalities. She continued to do well on hospital day two and was discharged on a beta-blocker and dual antiplatelet therapy, with counseling to avoid estrogen-containing oral contraceptives and to strictly abstain from cannabis; cardiac rehabilitation follow-up was arranged for activity guidance.

**Figure 3:**
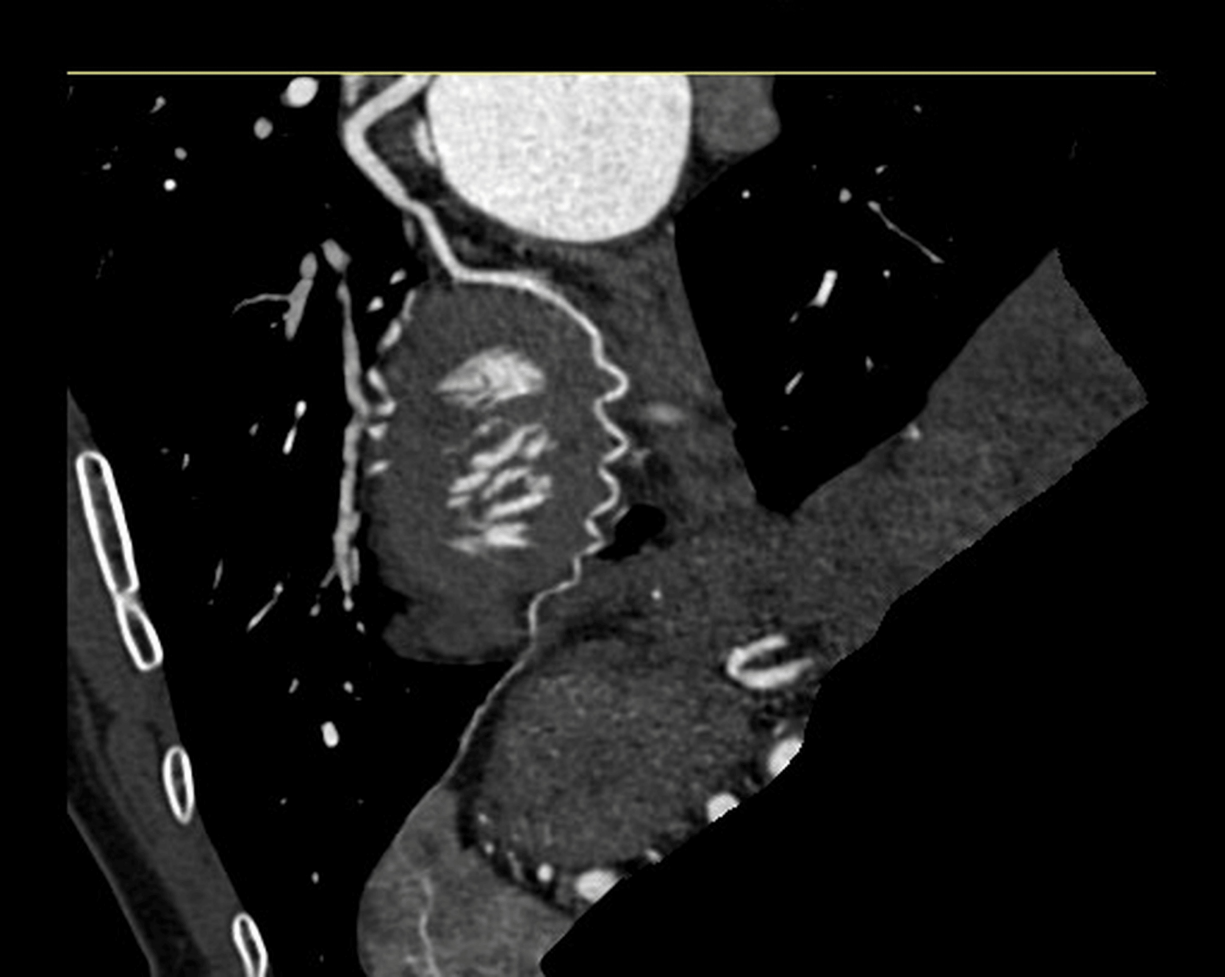
Follow-up CT coronary angiography of the LCx artery Curved planar reformatted CT coronary angiography at six-month follow-up demonstrated a patent LCx artery with interval resolution of the previously noted distal narrowing, consistent with spontaneous healing after conservative management of SCAD. LCx: left circumflex; SCAD: spontaneous coronary artery dissection

She had close follow-up at one, four, eight, and 12 weeks; her only ongoing symptom was mild exertional chest discomfort that gradually improved with activity modification. By 12 weeks, she was free of chest pain and reported abstinence from smoked cannabis. At three months, transthoracic echocardiography revealed a LVEF of 55%-60% and resolution of wall-motion abnormalities. CT coronary angiography at six months demonstrated resolution of the prior distal LCx abnormality, consistent with healing (Figure 3).

## Discussion

SCAD is defined as a dissection in the epicardial coronary arteries that is nontraumatic, noniatrogenic, and not due to atherosclerotic plaque rupture [1,2]. SCAD leads to ACS due to the sudden formation of an intramural hematoma (IMH) within any of the layers of the coronary artery wall (intima, media, or adventitia), ultimately leading to true lumen compression and vessel occlusion. Despite Pretty’s original description in 1931, the inciting event is still not fully elucidated [3,4].

Two mechanisms have been proposed: the inside-out and the outside-in [5]. The inside-out mechanism describes an intimal disruption allowing luminal blood to enter the vessel wall and create a false lumen, resulting in coronary dissection and IMH formation [6]. The outside-in mechanism proposes primary hemorrhage within the media (e.g., from the vasa vasorum), producing an IMH that expands and compresses the true lumen, leading to myocardial ischemia; secondary intimal rupture may occur later with decompression into the true lumen [7].

The incidence of SCAD in patients with ACS, based on a series of studies that meticulously excluded atherosclerotic, traumatic, and iatrogenic causes, is estimated at approximately 0.1%-4% of all ACS presentations overall [8-11]. It is well described that SCAD is disproportionately observed in women ≤50 years old, without traditional atherosclerotic risk factors [8-10]; one study even estimates that SCAD may account for up to 35% of all ACS in female patients ≤50 years of age [12] and is a leading etiology of pregnancy-associated myocardial infarction (MI) [13,14]. Given the unique profile of SCAD and the low prevalence of traditional risk factors, SCAD has been reported in association with arteriopathies such as FMD [15-17], inflammatory/immunologic diseases, the peripartum period, hormonal influences [18,19], and, more recently, cannabis exposure [20-22]. Causality is difficult to establish because background prevalence varies and screening is inconsistent across cohorts.

**Figure 4:**
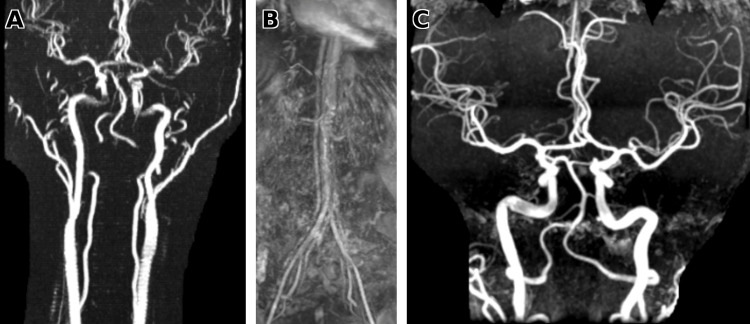
Extracoronary vascular imaging No features of FMD (beading, stenosis, or dissection) were noted. (A) Neck MRA (cervical carotid and vertebral arteries). (B) Renal artery MRA. (C) Brain MRA (intracranial arteries) without aneurysm or FMD-related changes. FMD: fibromuscular dysplasia; MRA: magnetic resonance angiography

The two most frequently reported associations in contemporary cohorts are FMD (25%-86%) [17,19] and exposure to exogenous hormones (10%-17%) [15]. FMD is a nonatherosclerotic, noninflammatory arteriopathy that most commonly involves the renal and cervicocerebral arteries but can affect any vascular bed; medial fibroplasia may produce the characteristic "string-of-beads" appearance on vascular imaging [18]. Because SCAD often coexists with extracoronary arteriopathy, vascular imaging beyond the coronaries is recommended when feasible. In our patient, we prioritized screening for FMD given its strong association with SCAD; extracoronary vascular imaging of the head/neck and renal arteries was negative (Figure 4). She had discontinued oral contraceptives one month prior to presentation, making a proximate hormonal trigger less likely.

In contrast, the link between symptoms and smoked cannabis exposure was striking: she experienced recurrent chest pain while actively smoking earlier that day and reported heavy use the morning of presentation, making cannabis the most plausible precipitating trigger in this case. This exposure-symptom clustering is the key clinical signal and the main reason this report should raise clinician awareness. Although the mechanism is uncertain, proposed contributors include sympathetic stimulation with increased blood pressure/heart rate (increased shear stress) and vasomotor effects, which could plausibly precipitate dissection in a susceptible vessel [20-22]. Published evidence remains limited to case reports [23-25], and many reports do not document extracoronary arteriopathy screening or follow-up coronary imaging, unlike our case. Prior published cases of cannabis-associated SCAD/coronary dissection are summarized in Table 1.

**Table 1:** Cannabis-associated SCAD cases Summary of published cannabis-associated SCAD/coronary dissection cases and key diagnostic elements compared with the present case. SCAD: spontaneous coronary artery dissection; FMD: fibromuscular dysplasia; CCTA: coronary CT angiography; LAD: left anterior descending; LCx: left circumflex; PCI: percutaneous coronary intervention; OCT: optical coherence tomography; IVUS: intravascular ultrasound; UDS: urine drug screen; THC: tetrahydrocannabinol; RCA: right coronary artery; CABG: coronary artery bypass graft; TIMI III: Thrombolysis in Myocardial Infarction grade III; EF: ejection fraction

Report	Cannabis exposure	Coronary findings (culprit)	OCT/IVUS	Extracoronary imaging (FMD screen)	Follow-up coronary imaging	Management/ outcome
Present case	Smoked; symptoms while smoking	Distal LCx long-segment high-grade narrowing	Not performed	Yes - negative (head/neck + renal)	Yes - CCTA healing (resolution)	Conservative; EF recovered, LCx healing
Arshad et al., 2023 [23]	Chronic marijuana; UDS THC+	LAD; angiography suggestive of dissection	None reported	None reported	None reported	PCI; outcome none reported
Filali et al., 2013 [24]	Smoked → daily heavy use × 1 month; UDS+	Multivessel dissection: LAD + LCx + RCA	None reported	None reported	None reported	CABG; short-term course uneventful
Ibn Hadj Amor et al., 2021 [25]	Smoked cannabis → daily × 5 days	Proximal LAD; Type 1 SCAD + thrombus; TIMI III	None reported	None reported	None reported	Conservative; 3 months asymptomatic

Invasive coronary angiography is the current gold-standard investigation. Saw [26] detailed the current classification of SCAD based on angiographic appearance: (i) Type 1, or "pathognomonic" appearance of multiple lumens and arterial wall contrast staining, is present in a small proportion of cases [27]; (ii) Type 2 refers to diffuse stenosis that can vary in length, bordered by normal segments proximal and distal to the stenosis, and is the most common type [27]; and (iii) Type 3 refers to short, tubular stenosis that can mimic atherosclerosis [27]. A "Type 4" pattern - complete coronary occlusion - was later proposed by Al-Hussaini and Adlam [28]. It is well described how difficult it is to differentiate SCAD Type 3 and Type 4 from atherosclerosis and how adjunctive intracoronary imaging, such as OCT or IVUS, can increase diagnostic confidence when available.

Saw et al. [29] characterized angiographic patterns in suspected SCAD, with OCT correlation in a subset, describing tortuosity, irregular stenosis, smooth long-segment narrowing, and ectasia. Notably, coronary tortuosity was present in 100% of cases in their cohort. In the present case, angiography localized the culprit to the distal LCx, a long-segment distal narrowing without an intimal flap or contrast staining, most consistent with probable SCAD within the Type 2/Type 3 spectrum (Video 1). Although intracoronary imaging would have increased mechanistic certainty, OCT was unavailable and IVUS was not suitable for distal LCx assessment; therefore, we anchored diagnostic confidence on angiographic morphology and clinical context, supported by minimal atherosclerotic burden elsewhere.

**Video 1:** Coronary angiography Coronary angiography in the RAO caudal view demonstrated a long-segment high-grade narrowing of the distal LCx artery beyond the first obtuse marginal branch. The clip shows delayed distal opacification and collateral filling. Findings are consistent with probable SCAD. RAO: right anterior oblique; LCx: left circumflex; SCAD: spontaneous coronary artery dissection

At the time of angiography, the principal alternative diagnoses included atherothrombotic ACS (plaque rupture with thrombus), coronary embolism, and vasospasm. Plaque rupture was considered less likely given the patient’s age, lack of traditional risk factors, and the absence of significant atherosclerotic disease in other epicardial vessels. Coronary embolism was less favored because the lesion appeared as long-segment distal narrowing rather than an abrupt focal cutoff, and no clear embolic source was identified during hospitalization. Vasospasm remained a consideration; however, the persistent high-grade distal LCx narrowing with delayed distal opacification via collateral filling was less typical for transient spasm alone. Taken together, the angiographic morphology and clinical context most strongly supported probable SCAD.

Our decision not to intervene was mainly based on the clinical pathway and risk factors. There were no signs of significant atherosclerosis in any other vessel, and the coronary angiographic appearance of the RCA (Figure 2A) and LAD artery (Figure 2B) was consistent with severe tortuosity, defined by Jakob et al. as the presence of ≥3 consecutive curvatures of 90°-180° in a major epicardial artery at end-diastole, and later classified by Eleid et al. as mild, moderate, or severe [30,31]. In addition to the suggestive angiographic findings, she remained hemodynamically stable with a preserved/low-normal ejection fraction and no significant mitral regurgitation [32].

Contemporary guidance favors conservative management in stable SCAD because many dissections heal spontaneously, whereas percutaneous coronary intervention can be technically challenging and carries higher risks of iatrogenic extension, stent malapposition, or failure to seal an IMH. In observational cohorts [33,34], approximately 83%-86% of patients were initially treated conservatively, and only ~2%-3% subsequently required revascularization due to extension or recurrent ischemia. Both support an invasive approach primarily in the setting of hemodynamic instability, ongoing ischemia, or high-risk anatomy (e.g., left main or proximal multivessel involvement).

A notable feature of this case was discordance between ECG localization, echocardiography, and coronary anatomy. The serial ECG pattern - anterolateral ST-segment depression with evolving precordial T-wave inversion - suggested anterior/anterolateral ischemia, typically raising concern for LAD-territory involvement. In contrast, transthoracic echocardiography demonstrated inferior wall hypokinesis, while angiography ultimately localized the culprit lesion to the distal LCx. This mismatch is clinically relevant because it may blur early culprit-vessel localization during ACS evaluation. We propose two possible explanations for these findings.

Recent studies help explain this discordance: unlike acute LAD or RCA occlusion, acute LCx occlusion often does not produce classic ST-elevation patterns on the standard 12-lead ECG [35,36]. Huey et al. reported that acute LCx occlusion demonstrated ST-segment elevation in 48% of cases, whereas 38% showed no ST-segment changes and 15% showed ST-segment depression [37]. These studies show that ECG findings in LCx ischemia depend on where the LCx is occluded and on coronary dominance (i.e., which artery supplies the posterior/inferior myocardium) [35,36]. In particular, lesions located distal to the first obtuse marginal branch and occurring in left-dominant or codominant systems are more likely to produce subtle ST-segment changes such as ST depression, rather than classic ST-elevation [36]. This mechanism aligns with our case given the distal LCx involvement.

A second possible explanation is subtle multivessel involvement. Multivessel SCAD has been described, and a second affected territory may be angiographically subtle, sometimes appearing only as mild diffuse narrowing [38,39]. Although the LAD artery was not identified as the primary culprit, two independent cardiologists reviewing the angiographic images raised the possibility of a subtle mid-to-apical LAD artery dissection. However, without adjunctive intracoronary imaging (OCT/IVUS), this could not be confirmed and may alternatively reflect nonspecific angiographic variation or dynamic vasomotor change. Accordingly, we present this only as a plausible hypothesis that underscores the limitation of angiography alone.

Compared with prior reports, this case provides a more complete workup of cannabis and temporally associated SCAD, with clear symptom timing around heavy smoked cannabis use, negative extracoronary screening for arteriopathy, and documented healing on follow-up CT coronary angiography (Table 1). In settings where intracoronary imaging is not available or not suitable for distal vessels, SCAD can still be approached responsibly by integrating angiographic morphology, minimal atherosclerotic burden elsewhere, and supportive follow-up imaging. Because she remained stable - no ongoing ischemia, preserved/low-normal LVEF, and no hemodynamic compromise - conservative management was appropriate. Overall, this case supports maintaining a high index of suspicion for SCAD in younger patients with ACS after cannabis exposure, and it highlights the value of trigger avoidance and close follow-up in resource-limited practice settings.

## Conclusions

SCAD is an uncommon cause of heart attack that often affects younger patients without typical cardiovascular risk factors. We report SCAD in a 39-year-old woman whose symptoms began while smoking cannabis and followed heavy use earlier that day. Unlike many published cannabis-related SCAD/dissection reports, our case includes negative extracoronary screening for FMD and clear healing on follow-up CT coronary angiography, strengthening diagnostic confidence and supporting cannabis as a possible, under-recognized trigger that warrants clinician awareness and further study.

## References

[1] Hayes SN, Tweet MS, Adlam D, Kim ES, Gulati R, Price JE, Rose CH (2020). Spontaneous coronary artery dissection: JACC state-of-the-art review. J Am Coll Cardiol.

[2] Waterbury TM, Tweet MS, Hayes SN (2018). Early natural history of spontaneous coronary artery dissection. Circ Cardiovasc Interv.

[3] Pretty HC (1931). Dissecting aneurysm of coronary artery in a woman aged 42. Br Med J.

[4] Alfonso F (2012). Spontaneous coronary artery dissection: new insights from the tip of the iceberg?. Circulation.

[5] Hayes SN, Kim ES, Saw J (2018). Spontaneous coronary artery dissection: current state of the science: a scientific statement from the American Heart Association. Circulation.

[6] Kaddoura R, Cader FA, Ahmed A, Alasnag M (2023). Spontaneous coronary artery dissection: an overview. Postgrad Med J.

[7] Dang Q, Burgess S, Psaltis PJ, Fairley S, Saw J, Zaman S (2024). Spontaneous coronary artery dissection: a clinically oriented narrative review. npj Cardiovasc Health 1.

[8] Nishiguchi T, Tanaka A, Ozaki Y (2016). Prevalence of spontaneous coronary artery dissection in patients with acute coronary syndrome. Eur Heart J Acute Cardiovasc Care.

[9] Mortensen KH, Thuesen L, Kristensen IB, Christiansen EH (2009). Spontaneous coronary artery dissection: a Western Denmark Heart Registry study. Catheter Cardiovasc Interv.

[10] Vanzetto G, Berger-Coz E, Barone-Rochette G (2009). Prevalence, therapeutic management and medium-term prognosis of spontaneous coronary artery dissection: results from a database of 11,605 patients. Eur J Cardiothorac Surg.

[11] Alfonso F, Bastante T (2014). Spontaneous coronary artery dissection: novel diagnostic insights from large series of patients. Circ Cardiovasc Interv.

[12] Saw J, Aymong E, Mancini GB, Sedlak T, Starovoytov A, Ricci D (2014). Nonatherosclerotic coronary artery disease in young women. Can J Cardiol.

[13] Manasrah N, Al Sbihi AF, Bell K, Afonso LC, Blank N (2021). Spontaneous coronary artery dissection: case series and literature review. Cureus.

[14] Vijayaraghavan R, Verma S, Gupta N, Saw J (2014). Pregnancy-related spontaneous coronary artery dissection. Circulation.

[15] Saw J, Ricci D, Starovoytov A, Fox R, Buller CE (2013). Spontaneous coronary artery dissection: prevalence of predisposing conditions including fibromuscular dysplasia in a tertiary center cohort. JACC Cardiovasc Interv.

[16] Poloskey SL, Olin JW, Mace P, Gornik HL (2012). Fibromuscular dysplasia. Circulation.

[17] Huart J, Stoenoiu MS, Zedde M, Pascarella R, Adlam D, Persu A (2023). From fibromuscular dysplasia to arterial dissection and back. Am J Hypertens.

[18] Evangelou D, Letsas KP, Korantzopoulos P, Antonellis I, Sioras E, Kardaras F (2006). Spontaneous coronary artery dissection associated with oral contraceptive use: a case report and review of the literature. Int J Cardiol.

[19] Mori R, Macaya F, Giacobbe F (2023). Association between hormone therapy and short-term cardiovascular events in women with spontaneous coronary artery dissection. Rev Esp Cardiol (Engl Ed).

[20] Goyal H, Awad HH, Ghali JK (2017). Role of cannabis in cardiovascular disorders. J Thorac Dis.

[21] Richards JR, Bing ML, Moulin AK, Elder JW, Rominski RT, Summers PJ, Laurin EG (2019). Cannabis use and acute coronary syndrome. Clin Toxicol (Phila).

[22] Seif El Dahan K, Machtoub D, Massoud G (2022). Cannabinoids and myocardial ischemia: novel insights, updated mechanisms, and implications for myocardial infarction. Curr Med Chem.

[23] Arshad H, Mousa A, Oudah B, Kakhktsyan T, Abu-Abaa M, Kass R (2023). Cannabis-induced ST-segment elevation myocardial infarction with possible coronary artery dissection: a case report. Cureus.

[24] Filali T, Lahidheb D, Gommidh M, Jdaida B, Hajlaoui N, Fehri W, Haouala H (2013). Spontaneous multivessel coronary artery dissection associated with cannabis use. J Cardiol Cases.

[25] Ibn Hadj Amor H, Touil I, Boukriba S, Bouchnak S, Kraiem S, Rouabhia R (2021). Case report: spontaneous simultaneous coronary and carotid dissection in a young cannabis user. F1000Res.

[26] Saw J (2014). Coronary angiogram classification of spontaneous coronary artery dissection. Catheter Cardiovasc Interv.

[27] Saw J, Starovoytov A, Humphries K (2019). Canadian spontaneous coronary artery dissection cohort study: in-hospital and 30-day outcomes. Eur Heart J.

[28] Al-Hussaini A, Adlam D (2017). Spontaneous coronary artery dissection. Heart.

[29] Saw J, Bezerra H, Gornik HL, Machan L, Mancini GB (2016). Angiographic and intracoronary manifestations of coronary fibromuscular dysplasia. Circulation.

[30] Jakob M, Spasojevic D, Krogmann ON, Wiher H, Hug R, Hess OM (1996). Tortuosity of coronary arteries in chronic pressure and volume overload. Cathet Cardiovasc Diagn.

[31] Eleid MF, Guddeti RR, Tweet MS (2014). Coronary artery tortuosity in spontaneous coronary artery dissection: angiographic characteristics and clinical implications. Circ Cardiovasc Interv.

[32] Estévez-Loureiro R, Lorusso R, Taramasso M, Torregrossa G, Kini A, Moreno PR (2024). Management of severe mitral regurgitation in patients with acute myocardial infarction: JACC focus seminar 2/5. J Am Coll Cardiol.

[33] Saw J, Humphries K, Aymong E, Sedlak T, Prakash R, Starovoytov A, Mancini GB (2017). Spontaneous coronary artery dissection: clinical outcomes and risk of recurrence. J Am Coll Cardiol.

[34] Saw J, Starovoytov A, Aymong E (2022). Canadian spontaneous coronary artery dissection cohort study: 3-year outcomes. J Am Coll Cardiol.

[35] Komatsu J, Nishimura YK, Sugane H (2022). Acute left circumflex coronary artery occlusion - diagnostic problems of initial electrocardiographic changes. Circ Rep.

[36] Geffin R, Triska J, Najjar S, Berman J, Cruse M, Birnbaum Y (2024). Why do we keep missing left circumflex artery myocardial infarctions?. J Electrocardiol.

[37] Huey BL, Beller GA, Kaiser DL, Gibson RS (1988). A comprehensive analysis of myocardial infarction due to left circumflex artery occlusion: comparison with infarction due to right coronary artery and left anterior descending artery occlusion. J Am Coll Cardiol.

[38] Salamanca J, García-Guimarães M, Sabaté M (2023). Multivessel spontaneous coronary artery dissection: clinical features, angiographic findings, management, and outcomes. Int J Cardiol.

[39] Lempereur M, Gin K, Saw J (2014). Multivessel spontaneous coronary artery dissection mimicking atherosclerosis. JACC Cardiovasc Interv.

